# Development and validation of an oligonucleotide microarray to characterise ectomycorrhizal fungal communities

**DOI:** 10.1186/1471-2180-9-241

**Published:** 2009-11-24

**Authors:** Marlis Reich, Annegret Kohler, Francis Martin, Marc Buée

**Affiliations:** 1 UMR 1136 INRA/Nancy Université Interactions Arbres/Microorganimes, INRA Nancy, 54280 Champenoux, France

## Abstract

**Background:**

In forest ecosystems, communities of ectomycorrhizal fungi (ECM) are influenced by several biotic and abiotic factors. To understand their underlying dynamics, ECM communities have been surveyed with ribosomal DNA-based sequencing methods. However, most identification methods are both time-consuming and limited by the number of samples that can be treated in a realistic time frame. As a result of ongoing implementation, the array technique has gained throughput capacity in terms of the number of samples and the capacity for parallel identification of several species. Thus far, although phylochips (microarrays that are used to detect species) have been mostly developed to trace bacterial communities or groups of specific fungi, no phylochip has been developed to carry oligonucleotides for several ectomycorrhizal species that belong to different genera.

**Results:**

We have constructed a custom ribosomal DNA phylochip to identify ECM fungi. Specific oligonucleotide probes were targeted to the nuclear internal transcribed spacer (ITS) regions from 95 fungal species belonging to 21 ECM fungal genera. The phylochip was first validated using PCR amplicons of reference species. Ninety-nine percent of the tested oligonucleotides generated positive hybridisation signals with their corresponding amplicons. Cross-hybridisation was mainly restricted at the genus level, particularly for *Cortinarius *and *Lactarius *species. The phylochip was subsequently tested with environmental samples that were composed of ECM fungal DNA from spruce and beech plantation fungal communities. The results were in concordance with the ITS sequencing of morphotypes and the ITS clone library sequencing results that were obtained using the same PCR products.

**Conclusion:**

For the first time, we developed a custom phylochip that is specific for several ectomycorrhizal fungi. To overcome cross-hybridisation problems, specific filter and evaluation strategies that used spot signal intensity were applied. Evaluation of the phylochip by hybridising environmental samples confirmed the possible application of this technology for detecting and monitoring ectomycorrhizal fungi at specific sites in a routine and reproducible manner.

## Background

Ectomycorrhizal (ECM) fungi form a mutualistic symbiosis with tree roots and play key roles in forest ecosystems. In return for receiving nutrients and water from the soil via the roots, they receive carbohydrates as photosynthate from their host plants [1]. As is the case for other soil fungal species, the composition of the ECM community is affected by both biotic and abiotic factors; these include climate changes, seasons, soil micro-site heterogeneity, soil and litter quality, host tree species and forest management [2-6]. To describe in more detail the impact of environmental factors on community composition, long-term, year-round monitoring and a detailed spatial description of the community has to be carried out. However, analyses are very often hindered by a limited sample number and by the ephemeral or cryptic lifestyle of the fungi [7,8].

Over the last fifteen years, PCR-based molecular methods and DNA sequencing of nuclear and mitochondrial ribosomal DNA have been used routinely to identify mycorrhizal fungi [9]. However, these methods are time-consuming and are limited in the number of samples that can be treated in a realistic time frame [10]. With automated molecular genotyping techniques, appropriate DNA databases [11] and a better knowledge of ITS variability within fungal species [12], identification of fungal taxa in environmental samples can now be expanded from the aforementioned methods to high-throughput molecular diagnostic tools, such as phylochips [13]. So far, DNA arrays have been mainly used for genome-wide transcription profiling [14,15], but also for the identification of bacterial species from complex environmental samples [16] or for the identification of a few genera of pathogenic fungi and Oomycetes [17,18]. Phylochips may comprise up to several thousand probes that target phylogenetic marker genes, such as 16S rRNA in bacteria or the internal transcribed spacer (ITS) region in fungi [19]; indeed, the latter is one of the most widely used barcoding regions for fungi [20]. Phylochips have several advantages over traditional approaches, including higher specificity, cost efficiency, rapid identification and detection of target organisms, and the high numbers of samples throughput; therefore, they are increasingly used for the detection of bacterial and pathogenic fungi [21,22]. In the ECM fungal ecology field, the first application of ribosomal DNA arrays was reported by Bruns and Gardes [23]; they developed a specific phylochip (on nylon membranes) to detect Suilloid fungi. Recently, this approach has also been used for truffle identification [24]. To the best of our knowledge, no study has reported the construction and application of an ECM fungal phylochip to detect a large number of ECM fungal species that belong to various genera from environmental samples.

Here, we report the first application of a custom ribosomal ITS phylochip to describe the community composition of ECM fungi on roots. The phylochip carried specific oligonucleotides for 95 fungal species that belong to 25 ECM fungal genera. The specificity of the oligonucleotides was evaluated using ITS amplicons of known reference species. The method was then used to describe ECM fungal communities that were obtained from 30-year-old spruce and beech plantations. To validate the phylochip, morphotyping and ITS sequencing of the ECM root tips, together with sequencing of ITS clone libraries, were carried out. We discuss the pros and cons of the phylochip in comparison to conventional approaches, and outline its potential applications for environmental monitoring.

## Results

### Identification of ECM fungi from environmental samples by morphotyping/ITS sequencing and sequencing of ITS clone libraries

By combining morphotyping and ITS sequencing of individual ECM root tips, and sequencing of ITS clone libraries, 26 fungal species were identified on the roots of beech and spruce trees; these included 25 ECM fungi (Table 1). Rarefaction curves of clone library coverage nearly reached a plateau, which indicated a near complete sampling of the ECM species in the soil samples that were taken from under the beech and spruce. In order to detect only one more species from spruce samples and a further two species from beech samples, it would be necessary to increase the sequencing effort two-fold (Additional file 1). The species richness was very similar for the two plantations, with 13 and 16 species being associated with spruce and beech, respectively; however, the community compositions were clearly distinct. Only three ECM taxa were found on the root tips of both hosts: *Cenococcum geophilum*, *Xerocomus pruinatus *and *Tomentellopsis submollis *(Table 1). Sequencing of the ITS clone libraries or identification of individual ECM morphotypes revealed similar fungal ECM profiles. Most fungi that were detected on spruce roots by sequencing of the ITS library were also detected by morphotyping (Additional file 2). Of these morphotypes, nine were also supported by sequencing the ITS of individual morphotypes (Table 1). One taxon was only identified with morphotyping and ITS-sequencing of individual ECM morphotypes, and another was identified only by morphotyping. Overall, 9 of 13 taxa (69%) from the spruce roots were identified by both molecular methods. A total of 10 of 16 taxa (62.5%) from the beech roots were identified by both approaches. Sequencing of the ITS clone libraries resulted in the detection of an additional two taxa. One of these was related to an unidentified endophyte, which was difficult to identify by morphotyping alone as it is likely leaving inside the root tissues (Table 1). A single taxon was identified only by the morphotyping/ITS sequencing approach, and three taxa were identified only by morphotyping. Using ITS1F and ITS4 primers [9] or NSI1/NLB4 [25], the ITS region from six ECM morphotypes (*Amanita rubescens, Inocybe sp 1, Lactarius sp 1 + 2, Tomentella sp 1, Tomentellopsis submollis*) were not amplified. The ITS regions from four fungi (*A. rubescens, Lactarius sp 1 + 2, Tomentella sp 1) *of those six morphotypes were also not amplified using the ITS clone library approach (Table 1). However, the use of the second primer pair, NSl1/NLB4, enabled the molecular biological characterisation of four morphotypes (*Piloderma sp., Sebacinaceae sp., Sebacina sp*. and *Pezizales sp*.) that were not amplified with ITS1f/ITS4.

**Table 1:** Fungal taxa identified on root tip samples from spruce and beech by sequencing of the ITS clone libraries of the pooled ECM tips and morphotyping/ITS sequencing of the individual ECM root tips.

	Pooled ECM tips ITS cloning/ITS sequencing		Individual ECM tips Morphotyping/ITS sequencing	
Species name	Acc. n°	Identities (%) (Unite^◆^/NCBI^○^)	Acc. n°	Identities (%) (Unite^◆^/NCBI^○^)
**ECM from *Picea abies***:				
*Thelephora terrestris*	EU427330.1	360/363 (100)^○^	UDB000971	142/151 (94)^◆^
*Cenococcum geophilum*	UDB002297	375/379 (98)^◆^	UDB002297	211/216 (97)^◆^
*Clavulina cristata*	UDB001121	375/375 (100)^◆^	UDB 001121	281/289 (97)^◆^
*Atheliaceae (Piloderma) sp*	AY097053.1	343/362 (94)^○^	EU597016.1	612/624 (98)^○^
*Cortinarius *sp 1	AJ889974.1	361/367 (98)^○^	UDB002224	232/242 (95)^◆^
*Xerocomus pruinatus*	UDB000018	348/351 (99)^◆^	UDB 000016	692/696 (99)^◆^
*Tomentelopsis submolli*s	AM086447.1	319/324 (98)^○^	morphotyping only	
*Inocybe *sp	AY751555.1	249/266 (93)^○^	morphotyping only	
*Xerocomus badius*	UDB000080	375/379 (98)^◆^	UDB000080	400/417 (95)^◆^
*Tylospora asterophora*	UDB002469	353/354 (99)^◆^	UDB002469	591/594 (99)^◆^
*Tylospora fibrillosa*	AF052563.1	405/408 (99)^○^	AJ0534922.1	561/578 (97)^○^
*Sebacina *sp	not detected		UDB000975	162/168 (96)^◆^
*Lactarius *sp 1	not detected		morphotyping only	
				
**ECM from *Fagus sylvatica***:				
*Pezizales sp*	UDB002381	28/28 (100)^◆^	DQ990873.1	602/646 (93)^○^
*Sebacinaceae sp*	EF619763.1	327/347 (94)^○^	EF195570.1	495/497 (99)^○^
*Laccaria amethystina*	UDB002418	356/360 (98)^◆^	UDB002418	276/277 (99)^◆^
Endophyte	AY268198.1	205/243 (84)^○^	not detected	
*Inocybe napipes*	UDB000017	292/294 (99)^◆^	UDB000017	148/155 (95)^◆^
*Xerocomus pruinatus*	UDB000483	241/242 (99)^◆^	UDB000483	279/288 (96)^◆^
*Cortinarius *sp 2	UDB002410	416/437 (95)^◆^	UDB002410	227/239 (95)^◆^
*Cortinarius *sp 3	UDB002170	306/316 (96)^◆^	UDB002445	57/59 (96)^◆^
*Cortinarius tortuosus*	UDB002164	279/284 (98)^◆^	not detected	
*Russula puellaris*	UDB000010	313/315 (99)^◆^	UDB000010	246/247 (99)^◆^
*Tomentellopsis submollis*	UDB000198	272/273 (99)^◆^	UDB000198	224/228 (98)^◆^
*Laccaria laccata*	UDB000104	322/327 (98)^◆^	UDB000769	283/283 (100)^◆^
*Cenococcum geophilum*	not detected		UDB002297	216/222 (97)^◆^
*Amanita rubescens*	not detected		morphotyping only	
*Lactarius sp 2*	not detected		morphotyping only	
*Tomentella sp*	not detected		morphotyping only	

For the sequence homology search, BLASTN was carried out with the NCBI (○) and UNITE (◆) databases. Accession numbers (Acc. n°) and identities are given.

### Specificity of designed oligonucleotides

**Figure 1:**
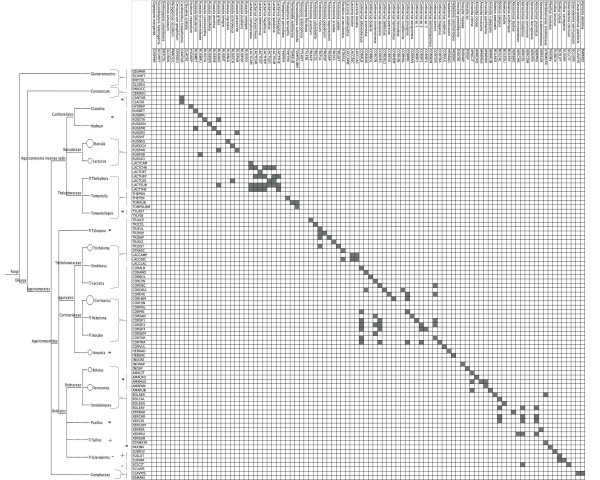
**Hybridisation reactions of the species-specific fungal oligonucleotides**. Reactions were tested by hybridising known fungal ITS pools to the phylochip. Vertical line indicates the fungal species used in the fungal ITS pools (hybridised probes), and the horizontal lines list the species-specific oligonucleotides. Grey boxes denote the positive hybridisation signals of an oligonucleotide obtained after threshold subtraction. The accompanying tree showing the phylogenetic relationship between tested fungal species was produced by the MEGAN programme. The size of the circle beside the genus name indicates the number of species of this genus used in the cross-hybridisation test.

The specificity of the 95 designed oligonucleotides (Additional file 3) was evaluated using PCR amplicons that were generated from sporocarp tissues. PCR amplicons mainly hybridised to the phylochip oligonucleotides according to the expected patterns (Figure 1), and the patterns were highly reproducible in the replications conducted with each of the templates. The hybridisation signal intensities ranged from -22 (background value) to 44,835 units. Ninety-nine percent of the oligonucleotides tested generated positive hybridisation signals with their matching ITS. Cross-hybridisations were mainly observed within the *Cortinarius *and *Lactarius *species complex. Among the *Boletaceae *species, a few cross-hybridisations were observed between the species that belonged to the *Boletus *and *Xerocomus *genera. Within the *Amanita*, *Russula *or *Tricholoma *genus, rare cross-reactions occurred between single sequences from closely related species.

### Identification of ECM species in root samples using phylochip

The ITS amplicons that were obtained from the two different environmental root samples were labelled and hybridised to the phylochips. The phylochip analysis confirmed the presence of most of the ECM fungi that were detected with the morphotyping, with the ITS sequencing of individual ECM tips, and with the ITS clone library approaches that were obtained using the same PCR products (Table 2). The exceptions included the following fungal species for which corresponding oligonucleotides on the phylochips were lacking: *Pezizales *sp, *Atheliaceae (Piloderma) *sp, *Sebacina *sp, *Sebacinaceae *sp, and unknown endophytic species. The inability to detect these four morphotyped ECM fungal species by molecular typing suggests that these morphological identifications could be incorrect (Table 2).

**Table 2:** Detection of fungal taxa from root tips of spruce and beech using different identification approaches.

Species name	Morphotyping/ITS sequencing of individual ECM tips	ITS cloning/sequencing of ECM tip pools	Phylochip
**samples from *Picea abies***			
*Thelephora terrestris*	x	x	x
*Cenococcum geophilum*	x	x	x
*Clavulina cristata*	x	x	x
*Atheliaceae (Piloderma) sp*	x	x	no oligonucleotide
*Cortinarius sp 1*	x	x	x
*Xerocomus pruinatus*	x	x	x
*Tomentellopsis submollis*	morphotyping only	x	x
*Inocybe sp*	morphotyping only	x	x
*Xerocomus badius*	x	x	x
*Tylospora asterophora*	x	x	x
*Tylospora fibrillosa*	x	x	x
*Sebacina sp*	x		no oligonucleotide
*Cortinarius sp 2*			x
*Russula integra*			x
*Cortinarius alboviolaceus*			x
*Cortinarius traganus*			x
*Amanita muscaria*			x
*Lactarius sp1*	morphotyping only		
			
**ECM from *Fagus sylvatica***			
*Pezizales sp*	x	x	no oligonucleotide
*Sebacinaceae sp*	x	x	no oligonucleotide
*Laccaria amethystina*	x	x	x
Endophyte sp.		x	no oligonucleotide
*Inocybe napipes*	x	x	x
*Xerocomus pruinatus*	x	x	x
*Cortinarius sp 2*	x	x	x
*Cortinarius sp 3*	x	x	x
*Cortinarius tortuosus*		x	x
*Russula puellaris*	x	x	x
*Tomentellopsis submollis*	x	x	x
*Laccaria laccata*	x	x	x
*Cenococcum geophilum*	x		x
*Cortinarius sp 1*			x
*Cortinarius hinnuleus*			x
*Russula integra*			x
*Laccaria bicolor*			x
*Amanita rubescens*	morphotyping only		
*Lactarius sp2*	morphotyping only		
*Tomentella sp*	morphotyping only		

Comparison of the abundance of sequences analysed by the cloning/sequencing approach and the species detection via the phylochip approach, indicated that the phylochip has the potential to detect taxa represented by approx. 2% of a DNA type in an environmental DNA sample. However, to assess the sensitivity of the current custom phylochip in more detail, further analyses will be carried out.

## Discussion

Many different environmental factors influence the dynamics and the spatiotemporal structure of ECM communities [26,27,5,4]. A better understanding of the mechanisms underlying these dynamics will require year-round ECM monitoring at incrementally increased spatial resolutions. However, the limited number of samples that can currently be analysed hinders the use of molecular approaches for large-scale studies. With the ongoing development of high-throughput molecular diagnostic tools, such as DNA oligoarrays [19] and 454 pyrosequencing [28], larger scale surveys (in terms of both the frequency and depth of analysis) of soil fungi are now possible. Ecologically relevant sample throughput in the in the 100 to 1000 range is now accessible. So far, phylochips have been used for the identification of bacteria [29], viruses [30], and a few genera of closely related fungal species [18].

In the present study, we constructed a custom ribosomal DNA phylochip for the identification of ECM fungi that was based on the ITS1 and ITS2 regions. One of the great advantages of using ITS regions for oligonucleotide design is the high number of sequences that are available in public databases [12]. Furthermore, these regions are some of the most frequently used regions for the barcoding of ECM fungi [20], and compared to other possible barcoding regions, they show a high specificity at the species level [31]. We designed a total of 95 oligonucleotides, from which 89 were species-specific for ECM fungal species. According to regular fruiting body surveys, these 89 ECM species are the most common species to be found in the long-term observatory of the Breuil-Chenue forest over the last ten years [32]. The ease with which high-quality species-specific oligonucleotides could be selected (mismatch in the middle of the designed oligonucleotide, without forming secondary structures), depended on the fungal genera. For example, the ITS sequences of *Laccaria *species showed only a few discriminative nucleotides that were spread as single nucleotide polymorphisms over the ITS1 and ITS2 regions. Consequently, prior to synthesis, oligonucleotide sequences were screened *in silico *for the presence of fortuitous similarities with fungal ITS sequences for which they were not designed.

The specificity of the spotted oligonucleotides was tested by hybridising ITS amplicons from reference species. Most of the oligonucleotides exhibited the expected hybridisation patterns (99% of the tested probes gave a positive signal with their corresponding ITS amplicon). However, cross-hybridisation was observed and it accumulated particularly in the genera *Cortinarius *or *Lactarius *that targeted other species in the same genus (Figure 1). With an estimated 2,000 spp. worldwide, *Cortinarius *is the most species-rich genus of mushroom-forming ECM fungi. Species delimitation within this genus is often controversial [33]. For these cryptic species, as for *Lactarius *or *Inocybe *species, the phylogenetic separation of species is ambiguous; indeed, most of these fungi have less than 3% intra-specific variability in the ITS region of their nuclear ribosomal DNA [34]. To keep cross-hybridisation low, we used a two-step data filtering process that involved: (i) accepting only spots with a significantly higher signal intensity value than the one obtained for the negative controls and, (ii) the requirement for a positive signal for at least four of the six replicates of one spot (see Methods). The hybridisation results were identical over the different replicates.

To test whether the current custom phylochip could be utilised in environmental studies that sought to describe the composition of an ECM community, ITS amplicons of root samples taken from beech and spruce plantations were hybridised to the array. As the focus of the current study was the validation of the phylochip, rather than an ecological study of the whole ECM fungal communities of the two plantations, a total of only six soil cores were used. The results of the phylochip were compared to the results that were obtained from the morphotyping/ITS-sequencing of individual ECM morphotypes and the sequencing of ITS clone libraries. Provided that the corresponding oligonucleotides were included on the array, all species that were detected by cloning-sequencing could also be identified with the phylochip. As the corresponding oligonucleotides were lacking on the phylochip, species belonging to the *Atheliaceae*, *Sebacinaceae *or *Pezizales *were not detected. Furthermore, the comparison of array signal intensity with ITS sequence frequency in the ITS clone library revealed the potential of the phylochip to detect taxa that were represented by approx. 2% of DNA types in the amplified DNA sample. However, the quantitative potential of this custom phylochip remains to be further accessed as bias linked to the PCR amplification could take place. The phylochip also detected species that were not expected according to the results obtained from the use of the other two approaches. This could be due to cross-hybridisations and/or to the fact that these under-represented species in the community could not be detected by the other approaches as the rarefaction curves of the ITS library sequencing method did not reach a plateau (Additional file 1).

When compared to each other, both of the other approaches provided similar, but not identical, profiles of the ECM communities. Approximately 70% of the species were detected using either method individually (Table 1). For the beech sample, three species were detected only by morphotyping as the PCR amplification of their DNA using ITS1F/ITS4 and/or NSI1/NLB4 primer pairs failed. Tedersoo et al. [35] showed that PCR of ITS from several ECM species failed using these universal fungal rDNA primers, and they stressed the need for additional taxon-specific PCR primers to be used for comprehensive genotyping of ECM communities. One of the morphotypes detected in the beech sample was a *Lactarius *species. In the same root sample, a *Pezizales *species was found by ITS-sequencing and cloning/sequencing; this suggests a possible co-colonisation of the ECM root tip [36]. ECM root tips can be colonised by more than one fungal taxon, by two different ECM species, or by one ECM species and an endophytic or parasitic species. Typically, these species are overlooked by the use of only morphotyping, but they can be detected by molecular biological approaches.

## Conclusion

In this study, we demonstrated that identification of ECM fungi in environmental studies is possible using a custom phylochip. The detection of most of the species by the phylochip was confirmed by two other widely used detection methods. Although the possible application of the phylochip technique to other study areas is dependent on the fungal species to be analysed, high-quality sequence support for several temperate and boreal forest ecosystems is found in databases such as UNITE [11]. For the next generation of phylochips, we will add additional species-specific probes or use additional marker gene regions in the probe design to overcome the small number of observed cross-hybridisations. In addition, we will increase the number of specific oligonucleotides that are spotted onto the phylochip (up to 10,000) to adapt to the taxonomic diversity found in soils at the study sites. Small-scale phylochips, so-called "boutique" arrays, such as the one designed in this study, are a time-saving and cheap approach for monitoring specific fungal species over years and/or in several hundred of samples. At the present time, the detection of a single species with our custom phylochip cost only one sixth of the price paid for the cloning/sequencing approach. The upscaling of detectable species on the phylochip (up to 10,000) will further lower the cost (by a factor of twenty). Thus, the phylochip approach should be an attractive method for routine, accurate and reproducible monitoring of fungal species on specific sites, in which a high sample throughput is required.

## Methods

### Site description and root sampling

The Breuil-Chenue experimental site is a temperate forest located in the Morvan Mountains (47°18'10"N, 4°4'44"E, France) at 650 m. The parent rock is granite and the soil is an alocrisol that is characterised by a pH ranging between 4 and 4.5, with moder type humus and micro-podzolisation features in the upper mineral horizon. In 1976, a part of the original stand, composed mainly of beech (90% of the stems), oak and young birch on a homogeneous soil type, was clear-cut. Subsequently, beech (*Fagus sylvatica L*.) and spruce (*Picea abies (L.) H. Karsten*) were planted separately in 20 m by 20 m adjacent stands [37].

Sampling of the root tips was performed in each stand (beech and spruce) in October 2007. A drill was used to obtain three soil cores (4 cm diameter × 10 cm depth) from each of the two treatments, along 18 m transects in the middle of each of the two plantations. The distance between the soil cores was 6 m, and the samples were collected at distances of more than 0.5 m from the trees or the stumps. Soil cores were immediately transported to the laboratory in isotherm boxes and stored at 4°C. Within five days, the roots were manually separated from the adhering soil, gently washed, and then examined under a stereomicroscope at 40×. Morphological typing of all of the ECM tips (approximately 50-250 tips per sample) was performed according to Agerer [38].

### ITS sequencing

**Figure 2:**
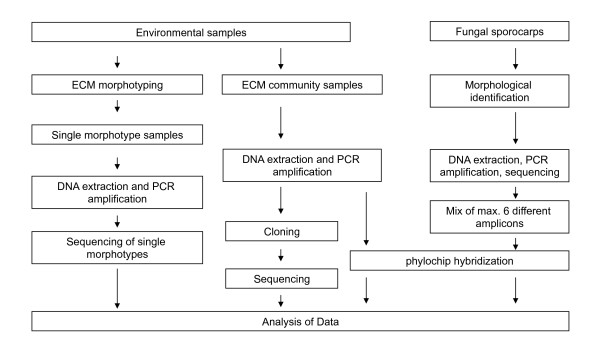
**The different procedures used for molecular genotyping of ECM root tips and evaluation of the phylochip**. DNA was extracted from individual ECM root tips or from pooled ECM root tips and subjected to PCR amplification to produce specific ECM ITS sequence or a heterogeneous mixture of ITS sequences, respectively. Individual ITS sequences were directly sequenced. The heterogeneous mixture of ITS sequences (ITS clone libraries) were either separated into individual molecules by cloning in bacterial plasmids or used directly for microarray hybridisation. The results of these three different technical approaches were analysed and compared. In addition, to test the specificity of the spotted oligonucleotides, the phylochips were hybridised with a heterogeneous mixture of ITS sequences from identified fungal sporocarps.

An individual ECM root tip from each ECM morphotype was selected for molecular characterisation by ITS sequencing. The remainders of the ECM root tips in each sample were used for ITS amplification, cloning and sequencing, and phylochip analysis (Figure 2). The samples were conserved at -20°C. DNA was extracted from single ECM root tips, or from the pooled ECM tips, and it was subjected to PCR amplification to produce a specific ITS amplicon or a heterogeneous mixture of ITS sequences (Figure 2), respectively. ITS amplicons from single tips were directly sequenced. Heterogeneous mixtures of sequences were either used to construct ITS clone libraries or used directly for phylochip hybridisation.

The ECM roots (up to 100 mg fresh weight depending on the sample) were freeze-dried and ground in a ball mill MM200 (Retsch^®^, Haan, Germany). Ground tissue was resuspended in 400 μl AP1 buffer from the DNeasy Plant Mini Kit (Qiagen, Courtaboeuf, France), and the DNA was extracted according to the manufacturer's instructions. Purified DNA was solubilised in dH_2_O (~100 ng/μl) and stored at -80°C. The ITS was amplified as described in Buée et al. [5], using primers ITS1F and ITS4 [9] and/or NSI1 and NLB4 [25]. PCR products were purified using a 96-well filtration system (MultiScreen-PCR plates, Millipore Corporation, MA, USA) and sequenced with ITS1F and/or ITS4 primers and the Genome Lab DTCS Quick Start Kit (Beckman Coulter, Roissy CDG, France), using a CEQ 8000XL sequencer and the CEQ 8000 Genetic Analysis System. ITS sequences were assembled with the Sequencher program for Macintosh, version 4.1.2 (Gene Codes Corporation, Ann Arbor, MI, USA), when sharing ≥ 97.0% identity. To identify the ECM fungi, BlastN was performed using ITS sequences that are available in the following public databases: NCBI http://www.ncbi.nlm.nih.gov/, UNITE http://unite.ut.ee/ and MycorWeb http://mycor.nancy.inra.fr/. ECM fungal morphotypes were considered to be identified at the species level when they shared ≥ 97% of their ITS region sequence identity with a sequence in these public databases [35].

### Sporocarp collection and taxonomic identification

Three times per year, during the autumnal periods of 2004 to 2007, fungal sporocarps of all epigeous fungi were surveyed at the Breuil-Chenue experimental site, and mature fungal fruiting bodies that exhibited all the characteristics necessary for an unequivocal identification, were collected. An expert mycologist, Jean Paul Maurice (Groupe Mycologique Vosgien, 88300 Neufchâteau, France), used traditional mycological methods for taxonomic determination of the sporocarps [39]. They were named according to the new "French Reference of Mycology" http://www.mycofrance.org. Samples were taken from the inner cap tissue (50-100 mg) and ground using a ball mill MM 200 (Retsch). DNA was extracted using the DNeasy Plant Mini Kit (Qiagen, Courtaboeuf, France) following the manufacturer's instructions. The ITS regions were amplified as described above, and they were used for hybridising the phylochips to assess the specificity of the designed oligonucleotides (see below).

### Cloning and sequencing of ITS

Prior to cloning, the amplified ITS products that were obtained from the bulk ECM tips of all soil cores were pooled to obtain only two samples: one sample each for the beech and spruce plantations. The amplified ITS fragments were cloned into *Escherichia coli *plasmids with the TOPO TA Cloning Kit, using the pCR^®^2.1-TOPO plasmid vector with a LacZα gene and One Shot DH5α chemically competent *Escherichia coli*, according to the manufacturer's instructions (Invitrogen, Cergy Pontoise Cedex, France). Seventy white recombinant colonies were selected; they were cultured overnight in LB medium and then frozen in glycerol at -80°C. Three microlitres of these bacterial suspensions were used directly for PCR, amplifying the inserts with M13-F (5'-GTAAAACGACGGCCAG-3') and M13-R (5'-CAGGAAACAGCTATGAC-3') primers. PCR was performed using the following protocol: initial denaturation at 94°C for 3 min, followed by 30 cycles of 94°C for 1 min, 50°C for 30 s and 72°C for 3 min, with a final extension step at 72°C for 15 min. The PCR products were purified with MultiScreen HTS™ PCR filter plates (Millipore, Molsheim, France). Sequencing was performed with a CEQ 8000XL sequencer (as described above), in which the ITS1F and ITS4 primer pairs were used to obtain sequences with lengths of up to 600 bp that included the ITS1 region and part of the ITS2 region. Sequences were edited as described above. The sequences can be accessed in public databases using the accession number FN545289 - 545352. In addition, a rarefaction analysis was performed to measure the proportion of the estimated diversity that could be reached by sequence effort using the freeware software Analytic Rarefaction version 1.3 http://www.uga.edu/strata/software/Software.html.

### Design of specific ITS oligonucleotide probes

To design specific ITS oligonucleotide probes for 89 ECM species, 368 ITS sequences of 171 ECM fungal species (around 600 bp) were aligned with the MultAlin program [40]. To take into account intraspecific ITS variability and sequencing errors, several ITS sequences from a number of different species were used for the alignment. Single nucleotide polymorphisms and indels were identified by manual curation. The sequences, including the ITS1, 5.8S and ITS2 regions of the nuclear rRNA genes, were obtained from the public databases NCBI and UNITE. Perfectly matching oligonucleotides, 67 to 70 bases in length, were designed for each ITS sequence within the ITS1 or ITS2 regions. They were selected for optimal melting temperatures (Tm; 75°C ± 2.5°C) and GC content (45-55%) using the AmplifX 1.37 software http://ifrjr.nord.univ-mrs.fr/AmplifX. To enhance specificity, oligonucleotides that had selective nucleotides located in a central position were favoured. The specificity of the oligoprobes was first tested *in silico *by querying the oligonucleotide sequences against the UNITE and NCBI databases. An oligonucleotide was designed as a positive hybridisation control on the ITS region of *Arabidopsis thaliana*. Five additional 62- to 70-mer oligonucleotides that matched the LSU region of the *Glomeromycota *were used to measure the background signal resulting from unspecific hybridisation. To avoid cross-hybridisations with undescribed species or cryptic species, we did not use the ITS region of untargeted fungal groups as a negative control.

### Spotting of glass slide microarray and hybridisation conditions

The 95 species-specific oligonucleotides (see above) were spotted; one well was spotted with only hybridisation buffer. Solutions of species-specific oligonucleotides were adjusted to a concentration of 600 pM and printed in triplicate by Eurofins, MWG/Operon (Cologne, Germany) on slide arrays with an activated epoxide surface. Oligonucleotides were bound via their 5' ends on the coating layer of the glass surface (for details, see http://www.operon.com). Arrays were prehybridised using the OpArray Pre-Hyb solution (Eurofins, MWG/Operon) according to the manufacturer's instructions. PCR-generated amplicons (maximal 30 ng/μl) were labelled with Alexa Fluor^® ^555 dye (Invitrogen, Cergy Pontoise, France) using the BioPrime^® ^Plus Array CGH Indirect Genomic Labelling System Kit (Invitrogen) following the manufacturer's instructions. After the last purification step, labelled amplicons were concentrated with a vacuum concentrator centrifuge UNIVAPO 100 H (UNIEQUIP, Martinsried, Germany), and then dissolved in 7 μl sterile water. The sample hybridisation procedure followed Rinaldi *et al*. [41] and is fully described in sample series GSM162978 in the GEO at NCBI http://www.ncbi.nlm.nih.gov/geo/. Slide arrays were scanned using a GenePix 4000 B scanner (Axon-Molecular Devices, Sunnyvale, CA, USA) at a wavelength of 532 nm for the Alexa Fluor 555 dye. Fluorescent images were captured as TIFF files and the signal intensity was quantified by GenePix Pro 5.0 software (Axon-Molecular Devices).

### Specificity of oligonucleotides and validation of the phylochip

To validate the specificity of the designed oligonucleotides, PCR-amplified ITS fragments from the sporocarp tissues of known fungal species were hybridised (Figure 2). Prior to hybridisation, amplicons (5 ng/μl) from three to six different ITS amplicons were mixed in a 1:1 ratio. Species-specific ITS within a mix were chosen based on the *in silico *tested species phylogenetic distance (minimal 30% bp differences were observed between the oligonucleotides of one species and the ITS sequences of the other species in the mix). In total, 74 fungal species were probed via the fungal amplicon mixes. The PCR product that was amplified from the ITS region of *Arabidopsis thaliana *was added to all amplicon mixes (at a concentration of 5 ng/μl) as a positive hybridisation control. To test the possible use of this custom phylochip for describing ECM community composition in environmental samples, 10 μl of the PCR product that was amplified from the bulked ECM root tips of beech and spruce was used (spiked with the amplicon of *Arabidopsis thaliana)*. Six technical replicates were carried out for each sample (three block replications per slide × two slides per sample). The results of the cross-hybridisation test are outlined in Figure 1. The ITS-based cladogram was constructed for all tested fungal species using the default setting of the MEGAN software (version 3.0.2., [42]).

### Array evaluation

Prior to further analyses, spots exhibiting poor quality (for example, as a result of the presence of dust) were flagged and excluded from the analyses. Hybridisation quality was surveyed using the positive (oligonucleotides of *Arabidopsis thaliana*) and negative controls (five oligonucleotides for the *Glomeromycota *(non-ECM species) and the one spot spotted with only hybridisation buffer) of each array. Data of the array were further used when (i) signal intensity values of the positive controls were within the group of oligonucleotides that showed the highest signal intensity values and (ii) the mean signal intensity value of the negative controls were a maximal 1.5% of the signal intensity with the highest value.

Individual spots were considered to be positive (species present in the sample) if their signal intensity showed a value that was five-fold higher than the averaged intensity value for all of the negative controls. Additionally, at least four of the six replicates per spot were required to generate a significant positive hybridisation. The threshold factor was fixed to five-fold after evaluation of the results of the arrays that were hybridised with the known amplicon mixes derived from sporocarp tissues (see "Sporocarp collection" and "Specificity of oligonucleotides"). Using a threshold factor of "5" defined the minimal 90% of all species in the amplicon mixes as positive and filtered most false-positives (cross-hybridisation).

## Authors' contributions

MR conceived and designed the array, set-up the clone library, acquired, analysed, and interpreted the data and drafted the manuscript. AK analysed and interpreted the array data. FM conceived and directed the project and drafted the manuscript. MB carried out the morphotyping and sequencing of the ECM root tips, drafted the manuscript and co-directed the project. All authors read and approved the final manuscript.

## Supplementary Material

Additional file 1 **Rarefied species accumulation curve of fungal species detected in ECM root tip samples of (A) spruce and (B) beech**. Figures of the rarefaction curves of detected fungal species in ECM root tips of spruce and beech. Click here for file

Additional file 2 **Species described by morphotyping with description of observed morphotypes according to Agerer (1987-2001)**. List of all ECM species detected by morphotyping and detailed description of their morphotypes. Click here for file

Additional file 3 **Sequences of the 95 species-specific oligonucleotides**. List of sequences of the 95 designed species-specific oligonucleotides. Click here for file
