# Supplementary material for: Development and validation of an oligonucleotide microarray to characterise ectomycorrhizal fungal communities
Source: BMC Microbiol. 2009 Nov 24;9:241. doi: 10.1186/1471-2180-9-241 (PMC2789087; doi:10.1186/1471-2180-9-241)

Additional file 1: Rarefied species accumulation curve of fungal species detected in ECM root tip samples of (A) spruce and (B) beech.

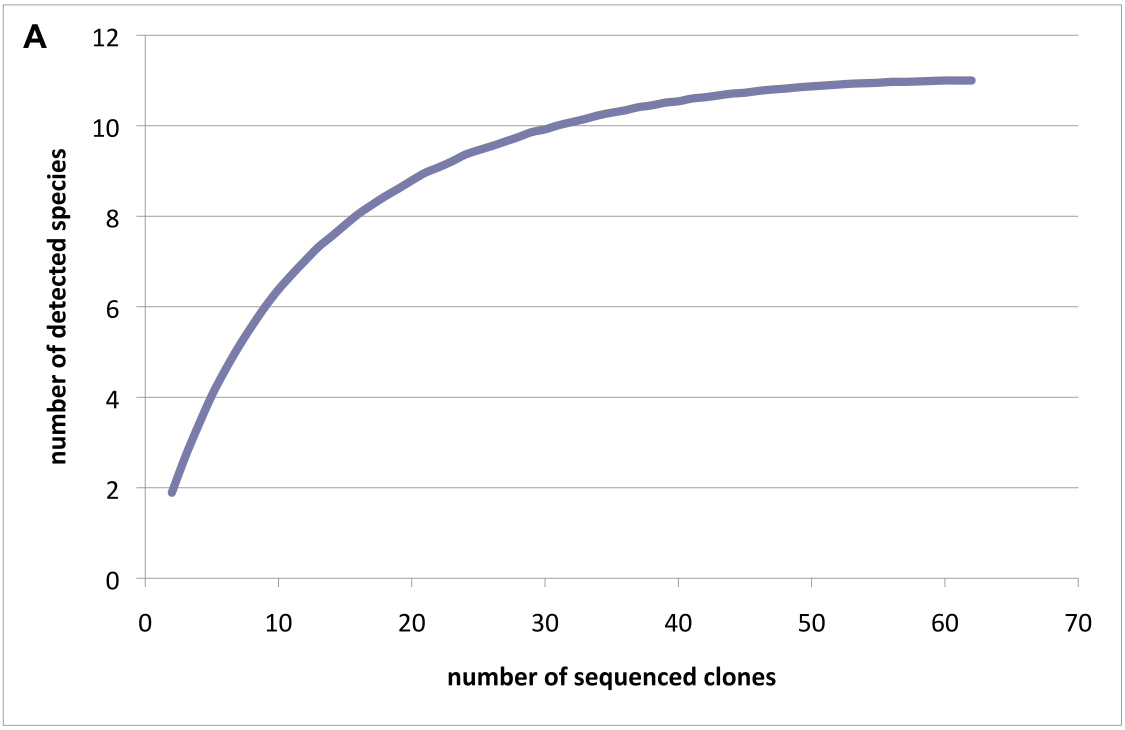

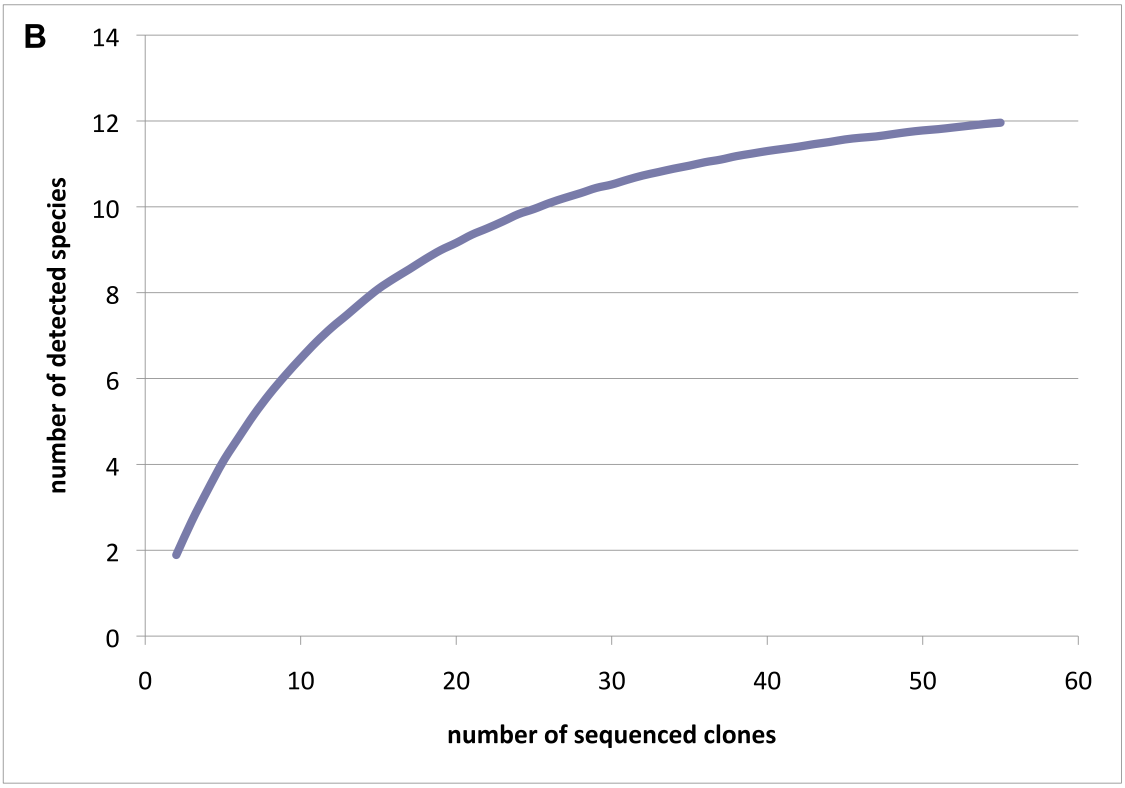

Supplement: Additional file 1 — Rarefied species accumulation curve of fungal species detected in ECM root tip samples of (A) spruce and (B) beech. Figures of the rarefaction curves of detected fungal species in ECM root tips of spruce and beech. [file 1471-2180-9-241-S1.PDF]
