# Supplementary material for: Development and validation of an oligonucleotide microarray to characterise ectomycorrhizal fungal communities
Source: BMC Microbiol. 2009 Nov 24;9:241. doi: 10.1186/1471-2180-9-241 (PMC2789087; doi:10.1186/1471-2180-9-241)
Supplement: Additional file 2 — Species described by morphotyping with description of observed morphotypes according to Agerer (1987-2001). List of all ECM species detected by morphotyping and detailed description of their morphotypes. [file 1471-2180-9-241-S2.PDF]

Additional file 2: Species described by morphotyping with description of observed morphotype according to Agerer (1987-2001).

ECM fungal species	Morphotyping
**ECM from *Picea abies***	
Cenococcum geophilum	Dark and shiny mycorrhizal mantle with adhering soil debris within hyphae
Clavulina cristata	Monopodial mycorrhizae with velvet white mantle
Cortinarius sp1	Tortuous mycorrhizal tips with emanting hyphae and white rhizomorphs
Inocybe sp	Monopodial mycorrhizae, silvery and velvet mantle (cystidia) coated by soil particules
Lactarius sp 1	Pyramidal mycorrhizae, smooth mantle with laticifers
Piloderma sp	Branched mycorrhizae with cottony hyphae and few pale rhizomorphs on yellow-brown mantle
Sebacina sp	Monopodial mycorrhizae with pearly white mantle
Thelephora terrestris	Monopodial-pinnate mycorrhizal system, smooth yellow-brown mantle
Tomentellopsis submollis	Tortuous mycorrhizal tips with white emanting hyphae and pinkish rhizomorphs
Tylospora asterophora	Monopodial-pyramidal mycorrhizae, yellow-white smooth mantle and emanating hyphae
Tylospora fibrillosa	Pyramidal mycorrhizae, yellow-white smooth mantle and cottony hyphae
Xerocomus badius	Branched mycorrhizae with rhizomorphs, shiny-yellow mantle (presence of air)
Xerocomus pruinatus	Monopodial-pyramidal mycorrhizae with rhizomorphs, white mantle (presence of air)
**ECM from *Fagus sylvatica***	
Amanita rubescens	Monopodial mycorrhizae with velvety claret-coloured mantle
Cenococcum geophilum	Dark and shiny mycorrhizal mantle with adhering soil debris within hyphae
Cortinarius sp 2	Tortuous mycorrhizal tips with cottony hyphae
Cortinarius sp 3	Tortuous mycorrhizal tips with emanting hyphae and white rhizomorphs
Inocybe napipes	Irregulary pinnate mycorrhiza, emanating hyphae enveloping mycorrhizal system
Laccaria amethystina	Monopodial mycorrhiza with purple tips and velvet surface mantle (emanating hyphae)
Laccaria laccata	Monopodial mycorrhiza with velvet honey-yellowish surface mantle
Lactarius sp 2	Pyramidal mycorrhiza, smooth honey-yellow mantle (laticiferous hyphae)
Pezizales sp	Dichotomous mycorrhizae with pale yellow mantle
Russula puellaris	Monopodial-pyramidal mycorrhizae, yellowish mantle with presence of air (light shiny)
Sebacinaceae sp	Dichotomous mycorrhiza, smooth greenish mantle
Tomentella sp	Monopodial-pinnate mycorrhiza with grainy brown mantle (few emanating hyphae)
Tomentellopsis submollis	Dichotomous mycorrhizae with several pink rhizomorphs on white mantle (with air)
Xerocomus pruinatus	Monopodial-pyramidal mycorrhizae with rhizomorphs, silvery-white mantle (presence of air)
