# Supplementary material for: Development and validation of an oligonucleotide microarray to characterise ectomycorrhizal fungal communities
Source: BMC Microbiol. 2009 Nov 24;9:241. doi: 10.1186/1471-2180-9-241 (PMC2789087; doi:10.1186/1471-2180-9-241)
Supplement: Additional file 3 — Sequences of the 95 species-specific oligonucleotides. List of sequences of the 95 designed species-specific oligonucleotides. [file 1471-2180-9-241-S3.PDF]

Additional file 3: Sequences of the 95 species-specific oligonucleotides.

Species	Abbreviation	Oligonucleotide Sequence (5’-3’)
Boletus aestivalis	BOLAES	GAGTGTCATGGAATTCTCAACCGTGTCCTCGATCTGATCTCGAGGCATGGCTTGGACTTGGGAGTTGCTG
Boletus calopus	BOLCAL	TTGAGTGTCATCGAATTCTCAACCATGTCTCGATCTATTTCAAGGCATGGCTTGGAGTTGGGGGTTTGCT
Boletus edulis	BOLEDU	CCTGAAATGCATTAGCGATGTTCAGCAAGCCTGAACGTGCACGGCCTTTTCGACGTGATAACGATCGTCG
Boletus erythropus	BOLERY	TTGAGTGTCATTTGAATTCTCAACCATGTCTTGATTGATTTCGAGGCATGGCTTGGACTTGGGGGTTGCT
Strobilomyces strobilaceus	STRMSTR	GCAAAGACGTCGGCTCTCCTCAAACGCATGAGCGGGACTAGCATGTCCGGACGTG
Xerocomus badius	XERBAD	GAGGATCTATGTATTTCATCATCACACCTATCGTATGTCTAGAATGTCATCGTCGACCACTGGGCGGCGA
Xerocomus chrysenteron	XERCHR	GTGCACGTCCACCTTTCTCTTACTCTCACACCTGTGCACACATTGTAGGTCCTCGAAAGAGGATCTATGT
Xerocomus cisalpinus	XERCIS	GAAAGCGGTCGGCTCTCCTGAAATGCATTAGCAAAGGACAGCAAGTCTGACGTGCACGGCCTTGACG
Xerocomus communis	XERCOM	GACGTGATAATGATCGTCGTGGGCTGAAGCGTCGGACATGCATCGATTGTCTTGTTTGCTTCCAAATCAC
Xerocomus ferrugineus	XERFER	CGTCCCCTCACCTTTTCTATCTACACACACCTGTGCACCTATTGTAGATCCCTCTCGAAAGAGAGGGAA
Xerocomus pruinatus	XERPRU	TTCGCTGTGCACGTCTTTCTTTTCGTCGACCTTTCTCTTACTCTCACACCTGTGCACACACTGTAGGT
Xerocomus subtomentosus	XERSUB	CTTTCTTCTTTCTTGGATGGAAAGTATGGCTTGGAGTTGGGAGTTGCTGGCAGAGACTGTCAGCTCTCC
Paxillus involutus	PAXINV	GCCTTTCCTTTGGAAGACCCTTTCTTCACACCCGTGCACACATTGTAGGTCTCCGCGAGGGGATCTATGT
Scleroderma citrinum	SCLCIT	GCATGTCTACAGAATGTCGTCCGTGGCGTCGGGCCACCGTAAACCATAATACAACTTTCAGCGATGGAT
Scleroderma verrucosum	SCLVER	GAGTGTCATCGAAACCTCAGACCGACCCTTCGACCCCGTCGGAGCTCGGTCTGGACTTGTGGGAGTCTGC
Suillus bovinus	SUIBOV	CAACTCCTCTCGATTGACTTCGAGTGGAGCTTGGATAGTGGGGGCTGCCGGAGACCTGAATATTCGTGTT
Suillus luteus	SUILUT	CGGAGACACTGGATTCGTCCAGGACTCGGGCTCCTCTTAAATGAATCGGCTCGCGGTCGACTTTCGACTT
Suillus variegatus	SUIVAR	GACCCGCGTCTTCATAAGCCCCTTCGTGTAGAAAGTCTATGAATGTTTTTACCATCATCGACTCGCGACT
Lactarius camphorata	LACTCAM	CGCTGGCTTTCAACGTTGTTGCACGCCGGAGCGTGTCCTCTCACATAACACAATCCATCTCACCCTTTGT
Lactarius chrysorrheus	LACTCHR	CCTCTCACATAATAATCCATCTCACCCTTTGTGCACCACCGCGTGGGCACCCTTTGGGATC
Lactarius deterrimus	LACTDET	CGCTGACTTTTTGAGACACAAAAGTCGTGCACGCCGGAGTGCGTCCTCTCACATAAAATCCATCTCACCC
Lactarius hepaticus	LACTHEP	GCAAGGGCTGTCGCTGACTCTATAAAGTCGTGCACGCCCGAGTGTGTCCTCTCACATAATAATCCATCTC
Lactarius quietus	LACTQUI	CTTCTAATCGTCTCAACCTTGCATCGAGACAAACGTCTGAGCGTGGCTCCCTTCCCTGGGAAACTCTCTC
Lactarius subdulcis	LACTSUB	GCTGTCGCTGACTCAAAGTCGTGCACGCCCGAGTGTGTCCTCTCACATAAATAATCCATCTCACCCTTTG
Lactarius theiogalus	LACTTHE	GTCGTGAAAACCTCAACCTCTTTGGTTTCTTCTGGGGACCAAAGCAGGCTTGGACTTTGGAGGCCTTTTG
Russula betularum	RUSBET	GGTCATTTTCGACCGCGGAAAGGATTTTGGACTTGGAGGCCTTTTGCTGGTTTCACCTTGAAGCGAGCTC
Russula brunneviolacea	RUSBRU	CTTTTTCTTTGACGAGAAAAGGAGTTTTGGACTTGGAGGTTCAATGCCCGCTTTCGGCATCGAAAGCGAG
Russula cyanoxantha	RUSCYA	CTTCAACCTTTCTTGGTTTCTTGACCGAGGAAGGCTTGGACTTTGGGGGTCTTTCATTGCTGGCCTCTTT
Russula densifolia	RUSDEN	GTCGTGAATTTCTCAAACCTTCTTGGTTTCTTGATCAAGAAGGCTTTGGACTTTGGAGGTCTTTGCCGGC
Russula emetica	RUSEME	CTCCTCCCAAATGTATTAGTGGGGTCTGCATTGTCGGTCCTTGGCGTGATAAGTTGTTTCTACGTCTTGG
Russula grisea	RUSGRI	GTGCATCACCGCGTGGGGCCCTTCTCTTTTCGGAGAGGGGGGTTCACGTTTTTACAAGAACGAACCATTA
Russula integra	RUSINT	CCCTTTTTGTTTGAAAAGGATTTTTGGACTTGGAGGTTCCATGCTCGCCTTTGCTTTTGAAGGTGAGCTC
Russula nigricans	RUSNIG	GTCGTGAAATTCTCAAACCTTCTTGGTTCCTTGACCAAGATGGCTTTGGACTTTGGAGGCGTTGTGCTGG
Russula ochroleuca	RUSOCH	CCTTTTTCTTTTTGGGAAAGGGTTTTTGGACTTGGAGGCTTTTTGCTGGCTTCACCTTGAAGTGAGCTCC
Russula parazurea	RUSPAR	CCTTGACGTGATAAGTTTGCTTCTACGTCTTGGGTTTCGCACTGTCGCACCGGAACCTGCTTCCAACCGT

Russula puellaris	RUSPUE	CCTTTTGTGCATCACCGCGTGGGTCCCCCTTTGCGGGAGGGCTCGCGTTTTCACATAAAACTTGATACAG
Russula violeipes	RUSVIO	GCGTGGGCCACCTTCTTTGGCTTGTTTCAAAGAGGTCGGTTCACGTTTTTACACACACACACCTTTATG
Amanita citrina	AMACIT	AGGTCCTTATGCAGCATGCAGGGAACTTTTGGACATTGGGAGTTGCTGGTCACTGATAAAGTGGCTGGCT
Amanita crocea	AMACRO	CCTGTGCACCGCCTGTAGACACTCTGTGTCTATGATATATGTCACACACACACACACAGTTGTTTTAGGC
Amanita muscaria	AMAMUS	GTCAAAACATGCACTTGAGTGTGTTTTGGATTGTGGGAGTGTCTGCTGGCTTTATGAGCCAGCTCTCCTG
Amanita pantherina	AMAPAN	CCTGAAAGACATTAGCTTTGGAGGGATGTGCCAAGTCGCTTCTGCCTTTCCATTGGTGTGATAGACG
Amanita rubescens	AMARUB	TGGGATTTTTGGACATTGGGAGTTGCCGGCTGCTGATAAAGTGGTGGGCTCTTCTGAAAAGCATTAGTTG
Laccaria amethystina	LACCAME	GGATACCTCTCGAGGCAACTCGGATTTTAGGGTCGCTGTGCTGTACAAGTCGGCTTTCCTTTCATTTCCA
Laccaria bicolor	LACCBIC	GCTTGGTTAGGCTTGGATGTGGGGGTTGCGGGCTTCATTAATGAGGTCGGCTCTCCTTAAATGCATTAGC
Laccaria laccata	LACCLAC	GGATACCTCTCGAGGCAACTCGGATTTTAGGATCGCCGTGCTGCACAAGTCGGCTTTCCTTTCATTTCC
Strobilurus esculentus	STRESC	CTTTGTACTCTTGTTGCTGTGTGCTGGCTTCTTCGGAAGTATGGTGCACGCTTGAGTGCAAGGGTCTTC
Tricholoma acerbum	TRIACE	AACCTTACTCAGCTTTCGCTAGTCGAGTTAGGCTTGGATATGGGAGTTTGTGGGCTTCTCGAAGTCGGCT
Tricholoma columbetta	TRICOL	CACTTTTATCGGTTGAATTAGGCTTGGATGTGGGAGTCTTTGCTGGCTTCGCAAGAGGTTGGCTCTCCTT
Tricholoma fulvum/ pseudonictitans	TRIFUL	CTACGCCATCATGTGAAGCAGCTTTAAATTGGGGTTGCTGCTCTCTAACAGTCTCTTTGGTGGGACAATT
Tricholoma populinum	TRIPOP	CCTAAAGTCGATCAGGCTTGGATGTGGGAGTTTGCGGGCTTTTCTAAAGTCGGCTCTCCTTAAATTT
Tricholoma saponaceum	TRISAP	CCTTTTCAGCATTTATGTTGATCAGGCTTGGATGTGGGAGTTTGCGGGCTTCTCAGAAGTCGGCTCTCCT
Tricholoma sciodes	TRISCI	GACTTGGAATATCTCTAGAGGCAACTCGGTTTTGAGGATTGCTGTGCGCAAGCCAACTTTCCTTACAC
Tricholoma ustale	TRIUST	CCTTTTCGGCTTTTTCTAAGTCGATTTAGGCTTGGATGTGGGAGTTTGCGGGCTTCTCTGAAGTCGGCTC
Cortinarius alboviolaceus	CORALB	CCTTCTCATTGCTGAGTGGTTTGGATGTGGGGGTTTGCTGGCCTCTTAAATGAGTTCAGCTCTCCTGAA
Cortinarius anomalus	CORANO	CTTCAGCTTTTGCTTGTTGAGTGTTGGATGTGGGGGGTCTTTTGCTGGCCTTTTTTTAGAGGTCAGCTTC
Cortinarius bolaris	CORBOL	CTCCACCTGTGCACCTTTTGTAGACCTGAATAGCTTTCTGAATGCTAAGCATTCAGGCTTGAGGATTGAC
Cortinarius cinnamomeus	CORCIN	CCAGGGTTTTTGACTTGTCGAGTGTTTGGATGTGGGGGTCTTTTGCTGGTCTCTTTTGAGGTCGGCTCCC
Cortinarius decipiens	CORDEC	GGGTTTGCTGGCCTTTTAAAAGGTTCAGCTCCTCTGAAATGCATTAGCAGAACAACCTTGCTCATTGGTG
Cortinarius delibutus	CORDELI	GAACAATTTGTTGACTGTTCATTGGTGTGATAATTATCTGCGCTATTGAACTGTGAGGCAAGTTCAGCTTC
Cortinarius evernius	COREVE	CTCCACCTGTGCACCCTTTGTAGACCTCCCAGGTCTATGTTGCTTCTTCATTTACCCCAATGTATGT
Cortinarius hemitrichus	CORHEM	CAAACCTTCTCTTTGTTGAGCGGTTTTGGATGTGGGGGTTTGCTGGCCTCTTAAAAAGGTTCAGCTCCTC
Cortinarius hinnuleus	CORHIN	CTAGGGAGCATGTGCACACCTTGTCATCTTTATATCTCCACCTGTGCACTTCTTGTAGGCCTTTCAGGT
Cortinarius multiformis	CORMUL	CTCCACCTGTGCACCTTTTGTAGACCTGGATATCTCTCTGAGTGCTTGCGCTCAGGTTTGAGGATTGATT
Cortinarius privignus	CORPRI	CTTCTCATTGCTGAGTGGTTTGGATGTGGTGGTTTGCTGGCCTCTTAAATGAGTTCAGCTCTCCTGAATG
Cortinarius sanguineus	CORSAN	CCTGTGCACCTTTTGTAGATCTGGATATCTTTCTGAATGCCTGGCATTCAGGTTTGGGGATTGACTTTGC
Cortinarius sp 1	CORSP1	GGGAGCATGTGCACGCCTTGTCATCTTTATATCTCCACCTGTGCACCTTTTGTAGACCCTTTCCAGGTCT
Cortinarius sp 2	CORSP2	CCTGATGGGTTGTTGCTGGTTCTCTGGGAGCATGTGCACACCTGTCATCTTTATATCTCCACCTGTGCAC
Cortinarius sp 2	CORSP3	GGGAGCATGTGCACGCCTGTCATCTTTATATCTCCACCTGTGCACTTTTGTAGACCTTCTGGGTCTATGT
Cortinarius semisanguineus	CORSEM	CTGGTCTCTTTTGAGATCGGCTCCCCTGAAATGCATTAGCGGAACAATTTGTTGACCCGTTCATTGGTG
Cortinarius tortuosus	CORTOR	GCATGTGCACACCTGTCATCTTTATATCTCCACCTGTGCACCCTTTGTAGACCTTCTCAGGTCTATGTTG
Cortinarius traganus	CORTRA	CAACCTTCTCTTGTTTGAGTGGTTTGGATGTGGGGGTTTGCTGGCTTCTTAAAAGGGTTCAGCTCCTCTG
Cortinarius vulpinus	CORVUL	CAACCTCTTCAGCTTTTGCTTGTTGAGCGTTGGATGTGGGGGGTCTGTTTTGCTGGTCTTCTCAGGTCAG
Hebeloma radicosum	HEBRAD	GCTTTTGTTGATACTGGCTTGGATATGGGGGTCTATTTTGCTGGCTTCTTTACAGATGGTCAGCTCCCC

Hebeloma sacchariolens	HEBSAC	CAGCTTTTGTTGATAACGGCTTGGATATGGGGGTTTTTTTTTGCTGGCTTCTTCACAGATGGTCAGCTCC
Inocybe griseolilacina	ISOGRI	GCTGTCCCTTCCTTTGGGTACGTGCACGCTTGTCATCTTTATTTCTACCCACTGTGCACATATTGTAGAC
Inocybe napipes	INONAP	CTGCTGGCTCTCCTCGGAGGGCATGTGCACGCTTGTTGTCCATTATTTCTCCCACTGTGCACAAATTGTA
Inocybe sp	INOSP	GGCACGTGCACGCCTGTTTTTATTTGCTTCTCCAACTGTGCACAAATATCGTAGACCTTAGCAAGGCCTA
Thelephora penicillata	THEPEN	AAATGAATCAGCTTGCCAGTCTTTGGTGGCATCACAGGTGTGATAACTATCTACGCTTGTGGTGGTC
Thelephora terrestris	THETER	CTCTGTAGTTCTATGGTCTGGGGGACCCTGTCTTCCTTCTGTGGTTCTACGTCTTTACACACACACTGTA
Tomentella sublilacina	TOMSUB	CTGGGGGACCCTGTCTTCCTGCCGTGGTTCTACGTCTTTACACACACTCTGTAATAAAGTCTTATGGAA
Tomentellopsis submollis	TOMPSUBM	GATCACGGAGCCCTGATGGGCAACGAATGCCCTCGTCTATGAATATTTTCACACACGCTCAAAGTATGAC
Cantharellus tubaeformis	CANTUB	CGGTCGCTTCCAATTGGGGGTTGACTCATAGGGGGTACATCTGTTTGAGGGTCATTTGTACCTTCTCAAA
Clavulina cristata	CLACRI	CACCTGTGCACATTTTTGAGGGAGTCTTGAGTTGGTTGCCGCTCTTGGGTGATTTTCTCACATTCCCTTA
Hydnum repandum	HYDREP	GGTATTCCGGGGAGCACACCTGTTCGAGTGTCATTGAAACTCTCAAATAAAGGTGGTTTTTGCAGACCAT
Clavariadelphus pistillaris	CLAVPIS	GAGGAGCATGCCTGTTTGAGTGTCGTGAATCTCTCTCAATCCCACCTCTGTGGGCTTGGATTTGGATG
Ramaria abietina	RAMABI	GCATTAGCGTTCCGCCGCGAGTTCGGTTTCGTAACGACGGTGTGATAAGTAACACTTTGACGCCGTCTGG
Tylospora asterophora	TYLAST	CCGAGCCCTTGAATCCCAAACACCACATGTGAACCCACCGTAGGCCTTCGGGCCTATGTCTTATCATATA
Tylospora fibrillosa	TYLFIB	CCCCCAACAAACACCGTGGGCCTTCGGGCCCGCGTATATTTACTCTGAATGTGTATAGAATGTAAACC
Cenococcum geophilum	CENGEO	GACGATTGACTCATGTTGCCTCGGCGGGCTCGCCCGCCAGAGGATACATCAAAAATCTTGTTTTAACGGT
Enthrospora colombiana	ENTCOL	TGATCACCTCGCCGTCGATAGCTTTGCTAACCTCGGTGGGATCTGATTAACTAGAGATTAGACTGATCGT
Gigaspora margarita	GIGMAR	CCTTGATAGATGTGATGTTTGGGGTCGAGGATTGCAACGGATACCCCTTCGGGGCTAGCCGCCTGATCT
Glomus sinuosum	GLOSIN	CGTGGTGTTGCTTTTGTGACGCTTCGGAATTGGGTCATCTTGATCCTTTGGGTTAAGAGACT
Paraglomus occultum	PAROCC	CACAAGTCCTCTGGAACGTGGCATCGTAGAGGGTGAGAATCCCGTCTCTGGTCGTTGTCTTGCAGGCA
Scutellospora heterogama	SCUHET	GTCAGCGTCGATTTTGGATATCATAAAATGATTGGGGGGAAGGTAGCTCCTTCGGGAGTGTTATAGCCCT
Arabidopsis thaliana	ARATHA	AGCTTTTATCTCGGTCTTGTCGTGCGCGTTGCTTCCGGATATCACAAAACCCCGGCACGAAAAGTGTCA
